# Anxiety and depression among students in a greek university amidst COVID-19 pandemic

**DOI:** 10.1192/j.eurpsy.2021.756

**Published:** 2021-08-13

**Authors:** E. Sazakli, M. Leotsinidis, M. Bakola, K.S. Kitsou, K. Argyropoulos, A. Konstantopoulou, A. Katsifara, P. Gourzis, E. Jelastopulu

**Affiliations:** 1 Department Of Public Health, Medical School, University of Patras, Patras, Greece; 2 Postgraduate Program Of Public Health, Medical School, University of Patras, Patras, Greece; 3 Department Of Psychiatry, School of Medicine, University of Patras, Patras, Greece

**Keywords:** Anxiety, Depression, Students, COVID-19

## Abstract

**Introduction:**

The coronavirus pandemic has challenged the world with an unprecedented situation. Social distancing, self or quarantine isolation, personal hand hygiene, self-protection, and the fear of becoming infected with the virus, come with a psychological fallout. The COVID-19 pandemic has affected students around the world, in terms of their education and lifestyle.

**Objectives:**

To investigate the impact of COVID-19 pandemic on the students’ mental health and well-being at the University of Patras, in Western Greece.

**Methods:**

An online questionnaire was prepared to collect responses from students during April 2020. Socio-demographic data, academic status, opinions about distance learning, changes in daily routine during the lockdown and anxiety and depression scores, according to the Greek version of the Hospital Anxiety and Depression Scale (HADS), were gathered.

**Results:**

The total number of responders was 2009, of which 67.3% women. During lockdown, the 68% of the students returned to their family home. Anxiety and depression scores were higher in students with a low income, poor self-rated health, not informed about COVID-19, not satisfied with distance learning and being annoyed at staying home. Prevalence of anxiety and depression was found to be 35.8% and 51.2%, ranging from 26.7% to 48.2% for anxiety and from 36.3% to 60.5% for depression in Health Sciences and Humanities and Social Sciences, respectively.

**Conclusions:**

Depression rates among university students in Greece were alarmingly high, denoting the impact of lockdown and changes in students’ life, due to the COVID-19 pandemic.

